# *catena*-Poly[[[diaqua­copper(II)]-{μ-4,4′-[1,4-phenyl­enebis(methyl­eneimino)]dibenzoato}] monohydrate]

**DOI:** 10.1107/S1600536808024379

**Published:** 2008-08-06

**Authors:** Qiu-sheng Wang, Jie Ouyang

**Affiliations:** a School of Chemistry and Chemical Engineering, Tianjin University of Technology, Tianjin 300191, People’s Republic of China

## Abstract

The asymmetric unit of the title polymeric compound, {[Cu(C_22_H_18_N_2_O_4_)(H_2_O)_2_]·H_2_O}_*n*_, contains a Cu ion situated on an inversion center, half of a centrosymmetric 4,4′-[1,4-phenyl­enebis(methyl­eneimino)]dibenzoate ligand, a coordin­ated water mol­ecule in a general position and an uncoordin­ated water mol­ecule situated on a twofold rotation axis. The distorted octa­hedral coordination geometry of the Cu^II^ ion is formed by six O atoms. The –NH– groups of the ligand are involved in intra­molecular N—H⋯O hydrogen bonds, while the water mol­ecules participate in the formation of a three-dimensional supra­molecular framework *via* inter­molecular O—H⋯O hydrogen bonds.

## Related literature

For properties of 4,4′-(1,4-phenyl­enebis(methyl­ene))bis­(aza­nedi­yl)dibenzoic acid and its ramifications, see: Yamaguchi *et al.* (1991 ▶); Imhof & Göbel (2000 ▶). For supra­molecular networks in related structures, see: Jing *et al.* (2006 ▶).
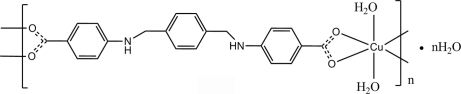


## Experimental

### 

#### Crystal data

[Cu(C_22_H_18_N_2_O_4_)(H_2_O)_2_]·H_2_O
*M* *_r_* = 491.98
Monoclinic, 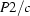

*a* = 16.127 (6) Å
*b* = 5.1535 (17) Å
*c* = 13.405 (8) Å
β = 92.76 (2)°
*V* = 1112.8 (8) Å^3^
*Z* = 2
Mo *K*α radiation
μ = 1.03 mm^−1^
*T* = 291 (2) K
0.09 × 0.08 × 0.07 mm

#### Data collection

Rigaku R-AXIS RAPID diffractometer
Absorption correction: multi-scan (*ABSCOR*; Higashi, 1995 ▶) *T*_min_ = 0.913, *T*_max_ = 0.932
10220 measured reflections
2534 independent reflections
2008 reflections with *I* > 2σ(*I*)
*R*_int_ = 0.040

#### Refinement

*R*[*F* ^2^ > 2σ(*F* ^2^)] = 0.043
*wR*(*F* ^2^) = 0.140
*S* = 1.03
2534 reflections
147 parameters
H-atom parameters constrained
Δρ_max_ = 0.49 e Å^−3^
Δρ_min_ = −0.49 e Å^−3^

### 

Data collection: *RAPID-AUTO* (Rigaku, 1998 ▶); cell refinement: *RAPID-AUTO*; data reduction: *CrystalStructure* (Rigaku/MSC, 2002 ▶); program(s) used to solve structure: *SHELXS97* (Sheldrick, 2008 ▶); program(s) used to refine structure: *SHELXL97* (Sheldrick, 2008 ▶); molecular graphics: *PLATON* (Spek, 2003 ▶); software used to prepare material for publication: *SHELXL97*.

## Supplementary Material

Crystal structure: contains datablocks global, I. DOI: 10.1107/S1600536808024379/cv2427sup1.cif

Structure factors: contains datablocks I. DOI: 10.1107/S1600536808024379/cv2427Isup2.hkl

Additional supplementary materials:  crystallographic information; 3D view; checkCIF report

## Figures and Tables

**Table 1:** Selected bond lengths (Å)

Cu1—O1	1.992 (3)
Cu1—O2	2.006 (2)
Cu1—O3	2.582 (2)

**Table 2:** Hydrogen-bond geometry (Å, °)

*D*—H⋯*A*	*D*—H	H⋯*A*	*D*⋯*A*	*D*—H⋯*A*
N1—H1⋯O3	0.84	2.00	2.676 (3)	137
O1—H1*A*⋯O3^i^	0.85	2.17	3.011 (3)	174
O1—H1*B*⋯O4^ii^	0.85	2.28	3.104 (3)	164
O4—H4*A*⋯O2^iii^	0.85	2.03	2.855 (3)	163

Symmetry codes: (i) 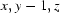; (ii) 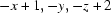; (iii) 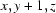.

**Fig. 1.**
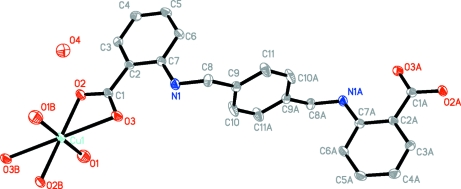
A portion of the polymeric chain in (I) with the atom numbering and 30% probalility displacement ellipsoids [symmetry codes: (A) 2-x, 1-y, 2-z; (B) 1-x, -y, 2-z]

## supplementary crystallographic information

### Comment

In recent years, 4,4'-(1,4-phenylenebis(methylene))bis(azanediyl)dibenzoic acid and its ramifications have become an area of interest owing to their various properties (Yamaguchi *et al.*, 1991; Imhof & Göbel, 2000). They are also used for building up supramolecular networks through hydrogen bonds (Jing *et al.*, 2006). Of special interest are the low-dimensional structural motifs related with highly anisotropic physical properties. Here we report the crystal structure of the title compound, (I).

For the title polymeric compound, (I), structure determination revealed a presence in the asymmetric unit of a half of centrosymmetric 4,4'-(1,4-phenylenebis(methylene))bis(azanediyl)dibenzoic ligand, one Cu ion lies on the inversion ctnter, one coordinated water molecule locates on the twofold axis and one lattice water molecule locates in the general positon. The Cu ion is coordinated by six oxygen atoms with four of which from 4,4'-(1,4-phenylenebis(methylene))bis(azanediyl)dibenzoic ligand and the other two from water moleculers into a distorted octahedral geometry (Table 1). The neighbouring Cu ions are linked by 4,4'-(1,4-phenylenebis(methylene))bis(azanediyl)dibenzoic ligand to form an infinite plolymeric zigzag chain (Fig. 1). The amino groups of the ligand are involved in intramolecular N—H···O hydrogen bonds, wihle water molecules participate in formation of three-dimensional supramolecular framework via intermolecular O—H···O hydrogen bonds (Table 2).

### Experimental

The 10 ml aqueous solution of CuCl_2_.2H_2_O (0.855 g, 5 mmol) was droped into a 10 ml DMF soution of 4,4'-(1,4-phenylenebis(methylene))bis(azanediyl)dibenzoic acid (1.882 g, 5 mmol). The mixture was stirred for half an hour. The resultant solution was filtered, and the filtrate was allowed to stand at room temperature for one week, to generate blue block crystals.

### Refinement

C-bound H atoms were geometrically positioned (C-H  0.93-0.97 Å) and treated as riding on their parent atoms, with *U*_iso_(H) = 1.2*U*_eq_(C) Water H atoms were positioned geometrically with O—H = 0.85 Å and *U*_iso_(H) = 1.2*U*_eq_(O). The N-bound H atom was located on a difference Fourier map, but placed in idealized position (N-H 0.84 Å) and refined as ridinh with *U*_iso_(H) = 1.5*U*_eq_(N).

### Crystal data

**Table:**

[Cu(C_22_H_18_N_2_O_4_)(H_2_O)_2_]·H_2_O	*F*_000_ = 510
*M**_r_* = 491.98	*D*_x_ = 1.468 Mg m^−^^3^
Monoclinic, *P*2/*c*	Mo *K*α radiation λ = 0.71073 Å
Hall symbol: -P 2yc	Cell parameters from 7833 reflections
*a* = 16.127 (6) Å	θ = 3.1–27.5º
*b* = 5.1535 (17) Å	µ = 1.03 mm^−^^1^
*c* = 13.405 (8) Å	*T* = 291 (2) K
β = 92.76 (2)º	Block, blue
*V* = 1112.8 (8) Å^3^	0.09 × 0.08 × 0.07 mm
*Z* = 2	

### Data collection

**Table:**

Rigaku R-AXIS RAPID diffractometer	2534 independent reflections
Radiation source: fine-focus sealed tube	2008 reflections with *I* > 2σ(*I*)
Monochromator: graphite	*R*_int_ = 0.040
Detector resolution: 10.0 pixels mm^-1^	θ_max_ = 27.5º
*T* = 291(2) K	θ_min_ = 3.0º
ω scans	*h* = −20→20
Absorption correction: multi-scan(ABSCOR; Higashi, 1995)	*k* = −6→6
*T*_min_ = 0.913, *T*_max_ = 0.932	*l* = −17→16
10220 measured reflections	

### Refinement

**Table:**

Refinement on *F*^2^	Secondary atom site location: difference Fourier map
Least-squares matrix: full	Hydrogen site location: inferred from neighbouring sites
*R*[*F*^2^ > 2σ(*F*^2^)] = 0.043	H-atom parameters constrained
*wR*(*F*^2^) = 0.140	*w* = 1/[σ^2^(*F*_o_^2^) + (0.0854*P*)^2^ + 0.6974*P*] where *P* = (*F*_o_^2^ + 2*F*_c_^2^)/3
*S* = 1.03	(Δ/σ)_max_ < 0.001
2534 reflections	Δρ_max_ = 0.49 e Å^−^^3^
147 parameters	Δρ_min_ = −0.49 e Å^−^^3^
Primary atom site location: structure-invariant direct methods	Extinction correction: none

### Special details

**Table:**

Geometry. All e.s.d.'s (except the e.s.d. in the dihedral angle between two l.s. planes) are estimated using the full covariance matrix. The cell e.s.d.'s are taken into account individually in the estimation of e.s.d.'s in distances, angles and torsion angles; correlations between e.s.d.'s in cell parameters are only used when they are defined by crystal symmetry. An approximate (isotropic) treatment of cell e.s.d.'s is used for estimating e.s.d.'s involving l.s. planes.
Refinement. Refinement of *F*^2^ against ALL reflections. The weighted *R*-factor *wR* and goodness of fit *S* are based on *F*^2^, conventional *R*-factors *R* are based on *F*, with *F* set to zero for negative *F*^2^. The threshold expression of *F*^2^ > σ(*F*^2^) is used only for calculating *R*-factors(gt) *etc*. and is not relevant to the choice of reflections for refinement. *R*-factors based on *F*^2^ are statistically about twice as large as those based on *F*, and *R*- factors based on ALL data will be even larger.

### Fractional atomic coordinates and isotropic or equivalent isotropic displacement parameters (Å^2^)

**Table:**

	*x*	*y*	*z*	*U*_iso_*/*U*_eq_	
C1	0.63113 (17)	0.1232 (5)	0.8986 (2)	0.0276 (6)	
C2	0.69938 (16)	0.1294 (5)	0.82720 (19)	0.0253 (5)	
C3	0.69767 (18)	−0.0493 (6)	0.7480 (2)	0.0312 (6)	
H3	0.6542	−0.1676	0.7419	0.037*	
C4	0.7583 (2)	−0.0552 (7)	0.6790 (2)	0.0383 (7)	
H4	0.7554	−0.1729	0.6264	0.046*	
C5	0.82372 (19)	0.1176 (7)	0.6898 (2)	0.0374 (7)	
H5	0.8653	0.1143	0.6441	0.045*	
C6	0.82859 (18)	0.2944 (6)	0.7668 (2)	0.0333 (6)	
H6	0.8733	0.4083	0.7724	0.040*	
C7	0.76652 (16)	0.3050 (5)	0.8376 (2)	0.0264 (5)	
C8	0.83674 (18)	0.6789 (5)	0.9238 (2)	0.0328 (6)	
H8A	0.8186	0.8126	0.9687	0.039*	
H8B	0.8427	0.7584	0.8590	0.039*	
C9	0.92123 (18)	0.5803 (6)	0.9623 (2)	0.0287 (6)	
C10	0.9299 (2)	0.3842 (8)	1.0310 (3)	0.0506 (9)	
H10	0.8826	0.3031	1.0530	0.061*	
C11	0.9926 (2)	0.6965 (7)	0.9314 (3)	0.0484 (9)	
H11	0.9888	0.8303	0.8848	0.058*	
Cu1	0.5000	0.0000	1.0000	0.02769 (18)	
N1	0.77292 (15)	0.4809 (5)	0.91439 (19)	0.0326 (5)	
H1	0.7305	0.4913	0.9484	0.049*	
O1	0.56996 (16)	−0.2511 (5)	1.07956 (19)	0.0517 (6)	
H1A	0.5868	−0.3765	1.0447	0.062*	
H1B	0.5446	−0.3136	1.1282	0.062*	
O2	0.57429 (13)	−0.0498 (4)	0.88572 (16)	0.0337 (5)	
O3	0.62970 (13)	0.2785 (4)	0.97072 (15)	0.0371 (5)	
O4	0.5000	0.5811 (7)	0.7500	0.0450 (8)	
H4A	0.5284	0.6994	0.7798	0.054*	

### Atomic displacement parameters (Å^2^)

**Table:**

	*U*^11^	*U*^22^	*U*^33^	*U*^12^	*U*^13^	*U*^23^
C1	0.0219 (13)	0.0338 (14)	0.0275 (14)	0.0043 (11)	0.0040 (10)	0.0018 (11)
C2	0.0218 (13)	0.0306 (14)	0.0237 (13)	0.0036 (10)	0.0039 (10)	0.0019 (10)
C3	0.0263 (14)	0.0380 (15)	0.0293 (15)	0.0004 (11)	0.0023 (11)	−0.0025 (11)
C4	0.0366 (17)	0.0494 (18)	0.0294 (15)	0.0018 (14)	0.0073 (12)	−0.0090 (12)
C5	0.0325 (16)	0.0504 (18)	0.0303 (15)	0.0016 (14)	0.0114 (12)	0.0013 (13)
C6	0.0259 (14)	0.0403 (16)	0.0345 (15)	−0.0032 (12)	0.0080 (11)	0.0032 (12)
C7	0.0222 (13)	0.0308 (13)	0.0264 (13)	0.0051 (10)	0.0016 (10)	0.0035 (10)
C8	0.0263 (14)	0.0307 (14)	0.0410 (16)	0.0006 (11)	−0.0027 (11)	−0.0008 (12)
C9	0.0269 (14)	0.0273 (12)	0.0318 (14)	−0.0003 (11)	0.0005 (11)	−0.0023 (11)
C10	0.0249 (16)	0.057 (2)	0.070 (2)	−0.0083 (15)	0.0041 (15)	0.0301 (18)
C11	0.0311 (16)	0.053 (2)	0.061 (2)	−0.0044 (15)	0.0001 (15)	0.0326 (17)
Cu1	0.0237 (3)	0.0304 (3)	0.0297 (3)	−0.00293 (19)	0.00892 (18)	−0.00458 (18)
N1	0.0225 (12)	0.0392 (14)	0.0365 (13)	−0.0030 (10)	0.0055 (10)	−0.0070 (10)
O1	0.0498 (15)	0.0521 (14)	0.0537 (15)	0.0052 (12)	0.0086 (12)	−0.0021 (11)
O2	0.0267 (10)	0.0391 (11)	0.0361 (11)	−0.0065 (8)	0.0102 (8)	−0.0065 (8)
O3	0.0335 (11)	0.0444 (12)	0.0346 (11)	−0.0045 (9)	0.0133 (8)	−0.0111 (9)
O4	0.048 (2)	0.0425 (16)	0.0444 (19)	0.000	0.0024 (15)	0.000

### Geometric parameters (Å, °)

**Table:**

C1—O3	1.257 (3)	C9—C10	1.370 (4)
C1—O2	1.284 (4)	C9—C11	1.379 (4)
C1—C2	1.493 (4)	C10—C11^i^	1.389 (5)
C2—C3	1.405 (4)	C10—H10	0.9300
C2—C7	1.413 (4)	C11—C10^i^	1.389 (5)
C3—C4	1.378 (4)	C11—H11	0.9300
C3—H3	0.9300	Cu1—O1^ii^	1.992 (3)
C4—C5	1.383 (5)	Cu1—O1	1.992 (3)
C4—H4	0.9300	Cu1—O2	2.006 (2)
C5—C6	1.376 (4)	Cu1—O2^ii^	2.006 (2)
C5—H5	0.9300	Cu1—O3	2.582 (2)
C6—C7	1.413 (4)	Cu1—O3^ii^	2.582 (2)
C6—H6	0.9300	Cu1—C1^ii^	2.646 (3)
C7—N1	1.372 (4)	N1—H1	0.8420
C8—N1	1.450 (4)	O1—H1A	0.8500
C8—C9	1.521 (4)	O1—H1B	0.8500
C8—H8A	0.9700	O4—H4A	0.8500
C8—H8B	0.9700		
			
O3—C1—O2	120.4 (3)	C9—C11—C10^i^	120.7 (3)
O3—C1—C2	121.4 (3)	C9—C11—H11	119.7
O2—C1—C2	118.2 (2)	C10^i^—C11—H11	119.7
C3—C2—C7	118.8 (2)	O1^ii^—Cu1—O1	180.00 (13)
C3—C2—C1	118.8 (2)	O1^ii^—Cu1—O2	91.02 (10)
C7—C2—C1	122.4 (2)	O1—Cu1—O2	88.98 (10)
C4—C3—C2	122.2 (3)	O1^ii^—Cu1—O2^ii^	88.98 (10)
C4—C3—H3	118.9	O1—Cu1—O2^ii^	91.02 (10)
C2—C3—H3	118.9	O2—Cu1—O2^ii^	180.000 (1)
C3—C4—C5	118.5 (3)	O1^ii^—Cu1—O3	89.98 (10)
C3—C4—H4	120.7	O1—Cu1—O3	90.02 (10)
C5—C4—H4	120.7	O2—Cu1—O3	55.75 (7)
C6—C5—C4	121.4 (3)	O2^ii^—Cu1—O3	124.25 (7)
C6—C5—H5	119.3	O1^ii^—Cu1—O3^ii^	90.02 (10)
C4—C5—H5	119.3	O1—Cu1—O3^ii^	89.98 (10)
C5—C6—C7	120.8 (3)	O2—Cu1—O3^ii^	124.25 (7)
C5—C6—H6	119.6	O2^ii^—Cu1—O3^ii^	55.75 (7)
C7—C6—H6	119.6	O3—Cu1—O3^ii^	180.0
N1—C7—C6	120.0 (3)	O1^ii^—Cu1—C1^ii^	89.14 (10)
N1—C7—C2	121.8 (2)	O1—Cu1—C1^ii^	90.86 (10)
C6—C7—C2	118.3 (2)	O2—Cu1—C1^ii^	152.03 (9)
N1—C8—C9	114.5 (2)	O2^ii^—Cu1—C1^ii^	27.97 (9)
N1—C8—H8A	108.6	O3—Cu1—C1^ii^	152.22 (7)
C9—C8—H8A	108.6	O3^ii^—Cu1—C1^ii^	27.78 (7)
N1—C8—H8B	108.6	C7—N1—C8	123.9 (2)
C9—C8—H8B	108.6	C7—N1—H1	114.5
H8A—C8—H8B	107.6	C8—N1—H1	120.0
C10—C9—C11	117.6 (3)	Cu1—O1—H1A	112.8
C10—C9—C8	122.3 (3)	Cu1—O1—H1B	112.1
C11—C9—C8	120.0 (3)	H1A—O1—H1B	108.2
C9—C10—C11^i^	121.7 (3)	C1—O2—Cu1	104.92 (17)
C9—C10—H10	119.1	C1—O3—Cu1	78.92 (16)
C11^i^—C10—H10	119.1		

Symmetry codes: (i) −*x*+2, −*y*+1, −*z*+2; (ii) −*x*+1, −*y*, −*z*+2.

### Hydrogen-bond geometry (Å, °)

**Table:**

*D*—H···*A*	*D*—H	H···*A*	*D*···*A*	*D*—H···*A*
N1—H1···O3	0.84	2.00	2.676 (3)	137
O1—H1A···O3^iii^	0.85	2.17	3.011 (3)	174
O1—H1B···O4^ii^	0.85	2.28	3.104 (3)	164
O4—H4A···O2^iv^	0.85	2.03	2.855 (3)	163

Symmetry codes: (iii) *x*, *y*−1, *z*; (ii) −*x*+1, −*y*, −*z*+2; (iv) *x*, *y*+1, *z*.
